# Multitasking Integrated Metasurface for Electromagnetic Wave Modulation with Reflection, Transmission, and Absorption

**DOI:** 10.3390/mi15080965

**Published:** 2024-07-28

**Authors:** Jiayun Wang, Yuanyuan Niu, Qiang Zhao, Yuxue Shang, Yuanhui Wang

**Affiliations:** 1 State Key Laboratory of Dynimic Measurement Technology, North University of China, Taiyuan 030051, China; 2 School of Instrument and Electronics, North University of China, Taiyuan 030051, China; 3 Shanxi Polytechnic College, Taiyuan 030006, China; 4 Institute for History of Science and Technology, Shanxi University, Taiyuan 030006, China; 5 Shanxi Lanhua Coal Industry Group Co., Ltd., Jincheng 048026, China; ariosozhao@gmail.com (Q.Z.); yuxue_shang@163.com (Y.S.); 6 School of Electrical and Control Engineering, North University of China, Taiyuan 030051, China; yhw@nuc.edu.cn

**Keywords:** metasurface, multitasking, EM modulation, reflection, transmission, absorption

## Abstract

Accommodating multiple tasks within a tiny metasurface unit cell without them interfering with each other is a significant challenge. In this paper, an electromagnetic (EM) wave modulation metasurface capable of reflection, transmission, and absorption is proposed. This multitasking capability is achieved through a cleverly designed multi-layer structure comprising an EM Wave Shield Layer (ESL), a Polarization Modulation Layer (PML), and a Bottom Plate Layer (BPL). The functionality can be arbitrarily switched by embedding control materials within the structure. Depending on external excitation conditions, the proposed metasurface can realize reflection-type co-planar polarization to cross-polarization conversion, transmission-type electromagnetically induced transparency-like (EIT-like) modes, and broadband absorption. Notably, all tasks operate approximately within the same operating frequency band, and their performance can be regulated by the intensity of external excitation. Additionally, the operating principle of the metasurface is analyzed through impedance matching, an oscillator coupling model, and surface current distribution. This metasurface design offers a strategy for integrated devices with multiple functionalities.

## 1. Introduction

The terahertz wave denotes an EM wave characterized by a wavelength ranging from 0.03 to 3 mm and a frequency spectrum spanning from 0.1 to 10 THz. It falls between microwave and infrared frequencies in the EM spectrum [1,2]. Due to its strong penetrability, large information capacity, high security, and strong maneuverability, it has a wide range of applications in long-distance communication, security imaging, radar detection, biomedicine, and other fields [3,4,5,6]. Terahertz wave has attracted more and more scholars’ attention. However, the development and application of terahertz technology are limited due to the lack of high-performance terahertz functional devices. As a two-dimensional artificial EM metamaterial, the metasurface can respond excellently to THz waves [7,8,9]. The design not only offers a straightforward and compact structure but also presents the advantages of a low profile, minimal loss, and enhanced efficiency [10]. The metasurface is capable of regulating the amplitude, phase, and polarization of EM waves, which is essential for overcoming the barriers faced by terahertz technology [11,12,13].

As electronic device development becomes increasingly precise, the demands for miniaturization, integration, and lightweight design have correspondingly become more stringent. Integrating multiple tasks within a tiny cell, where these tasks can freely switch without mutual interference, presents a significant challenge. To address this technical issue, researchers have explored various switchable devices or materials to regulate multiple tasks, such as MEMS [13], PIN diodes [14,15], varactor diodes [16], graphene [17,18,19,20,21], light-controlled semiconductors [22,23,24,25], phase-change materials [26,27,28,29,30], etc. Thanks to the excellent characteristics of these devices and materials, many multifunctional integrated devices have been developed [31,32,33,34,35,36,37,38,39]. For example, Qi et al. proposed a quadruple-function terahertz absorbing metasurface that can switch between single-frequency, dual-frequency, broadband, and ultra-broadband absorbing functions by adjusting the excitation conditions of vanadium dioxide (VO_2_) and graphene embedded in the metasurface structure [34]; Chen et al. designed a multifunctional terahertz metamaterial absorber that utilizes the metallic state of VO_2_ and the insulating state of graphene to achieve absorptive properties with circular dichroism or linear dichroism [38]; Zhang et al. proposed a multifunctional metasurface device that can produce broadband absorption and beam steering functions through the regulation of VO_2_ [39]. Additionally, the integrated absorbing–transmitting metasurface can possess both in-band transmission and out-of-band absorption characteristics, playing a significant role in reducing the radar cross-section of targets and suppressing EM interference [40,41,42]. For instance, Pan et al. achieved broadband absorption at 1.46–2.29 THz and 3.51–4.3 THz and transmission at 2.9–3.21 THz by forming a highly frequency-selective transmission window through the strong interaction between electric and magnetic dipole moments [41]. Wang et al. designed a thermally controlled multifunctional metamaterial absorber based on resistive films and VO_2_, realizing an absorption region at 3.31–10 THz and a transmission region near 5.15 THz [42]. However, the aforementioned metasurfaces with absorption, reflection, and transmission capabilities do not regulate EM waves effectively, and the operating frequency bands for different tasks are located in different positions. Therefore, there is an urgent need to design a metasurface that can effectively regulate EM waves and simultaneously possess reflection, transmission, and absorption operating modes.

In this paper, we employ a dual-layer metasurface design that incorporates VO_2_ and graphene as regulatory materials to achieve various EM wave control functions, including reflection, transmission, and absorption. Depending on different external excitation conditions, the metasurface can freely switch between multiple tasks. For the reflection-type polarization conversion mode, it can realize co-planar polarization to cross-polarization conversion (LTL) in the range of 1.75–3.55 THz and linear polarization to circular polarization conversion (LTC) in the range of 3.60–4.69 THz. In terms of absorption mode, it can achieve over 90% absorptivity of EM waves in the 1.34–3.82 THz range. As for the EIT-like mode, a transmission window can be generated in the range of 3.48–4.93 THz, with nearby EM waves being reflected. In addition, due to the contribution of the tunable materials, all functions can be controlled by adjusting the magnitude or polarization direction of the excitation conditions. The devised metasurface, leveraging the capability of multiple tasks that can be arbitrarily switched, is applicable across an expansive array of scenarios.

## 2. Design, Methods, and Multitasking

**Figure 1:**
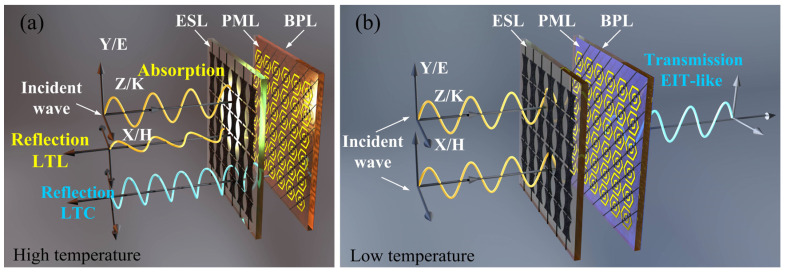
Schematics diagrams of the proposed multitasking metasurface. (**a**) high-temperature condition for reflection and absorption modes; (**b**) low-temperature condition for EIT-like mode.

The proposed multitasking metasurface consists of three layers: an EM Wave Shield Layer (ESL), Polarization Modulation Layer (PML), and Bottom Plate Layer (BPL), as illustrated in Figure 1. The ESL is positioned on the front side of Dielectric Layer 1 and consists of graphene hourglass-shaped resonators and longitudinal feed lines. These feed lines adjust the Fermi level *μ*_c1_ of graphene. Due to the anisotropy of the hourglass shape, the metasurface exhibits polarization-controllable characteristics in its task. The PML is situated on the front side of Dielectric Layer 2 and is composed of nested hexagonal rings, graphene gap blocks, and oblique feed lines designed to regulate the Fermi level *μ*_c2_ of graphene blocks. This configuration enables the tuning of the PML’s ability to manipulate the polarization properties of EM waves. The BPL layer is attached to the backside of Dielectric Layer 2 and is made up of a continuous VO_2_ plane. By controlling the phase of VO_2_, it regulates whether EM waves are allowed to pass through. When the metasurface is in operation, EM waves are incident on the front side of the metasurface, with the electric field polarized along the y-axis direction.

By utilizing the integrated graphene and VO_2_ within the structure, multiple tasks can be modulated by adjusting the Fermi level of graphene and the state of VO_2_. The multitasking operational modes of the designed metasurface and the corresponding external excitation conditions are illustrated in Figure 1 and Table 1. When the Fermi levels of graphene are uniformly set to 0 eV and VO_2_ is in the metallic state, the designed metasurface operates in polarization conversion mode. It achieves a linear polarization conversion efficiency of over 90% within the frequency range of 1.75–3.55 THz and facilitates right-hand circular polarization (RHCP) between 3.60 and 4.69 THz. By adjusting the Fermi level of graphene to 1 eV, the metasurface switches to absorption mode, attaining an absorptivity exceeding 90% in the range of 1.34–3.82 THz, as shown in Figure 1a. Furthermore, when the Fermi level *μ*_c1_ and *μ*_c2_ of graphene are set to 0 eV and 1 eV, respectively, and VO_2_ in the insulating phase, the metasurface engages in an EIT-like task, establishing a transmission window in the range of 3.48–4.93 THz, as shown in Figure 1b.

**Figure 2:**
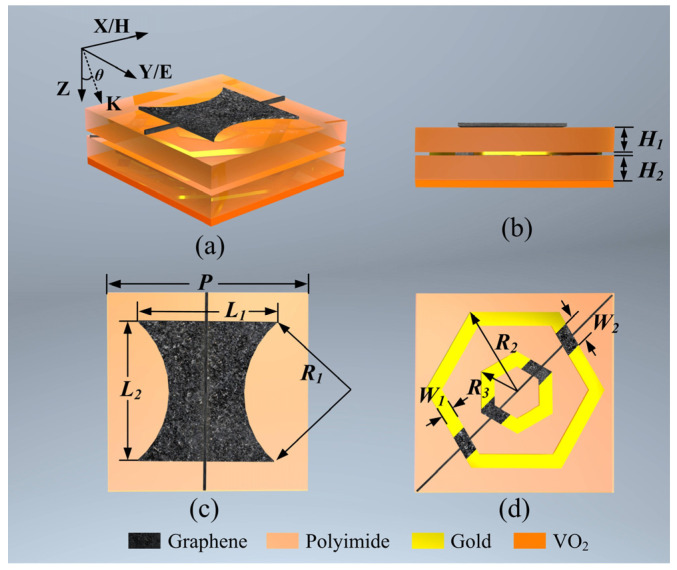
Schematics diagrams of the unit cells of the proposed multitasking metasurface. (**a**) perspective diagram of the unit cell; (**b**) side view of the unit cell; (**c**) top view of ESL; (**d**) top view of PML.

The detailed design of the metasurface unit cell is presented in Figure 2. Figure 2a,b show the overall view and side view of the unit cell, while Figure 2c,d show the front views of the ESL and PML. The dielectric layers are constructed using two layers of polyimide material, each with thicknesses of 9.88 μm and 6.03 μm, characterized by a permittivity and loss tangent of 3.5 and 0.0027, respectively. The nested hexagonal resonators within the PML layer are made of gold, with conductivity *σ*_Au_ of 4.561 × 10^7^ S/m. The metasurface’s other dimensional parameters were optimized through collaborative simulations using CST Studio Suite 2021 and MATLAB R2022a, leveraging the genetic algorithm solver from MATLAB’s Optimization Toolbox^TM^. The optimized structural parameters are summarized in Table 2.

**Figure 3:**
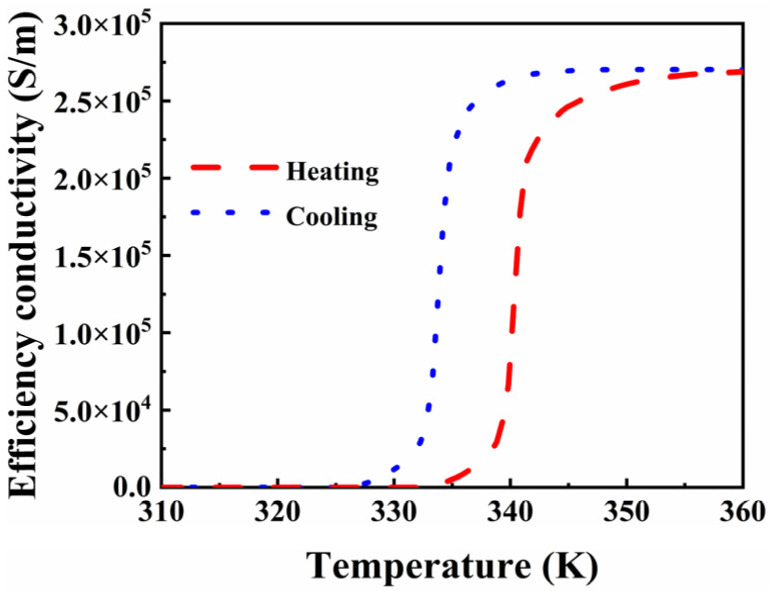
Efficiency conductivity of VO_2_ during heating and cooling.

To model and solve the metasurface, we employed the EM simulation software CST. In the simulation, boundaries with periodicity were established in the x- and y-axes, while transverse plane waves were sent perpendicularly into the unit cell along the negative z-axis. The entire simulation was carried out utilizing an adaptive fine mesh setting, subsequently simulating the corresponding values of the EM wave using a frequency domain solver. Furthermore, concerning the phase-change material VO_2,_ where *T*_0_ denotes the phase-change temperature, 68 °C and 62 °C signify the heating and cooling processes, respectively, displaying hysteresis loop Δ*T* = 6. Typically, the conductivity of VO_2_ is modulated by temperature. It initiates as an insulator at room temperature and shifts to a high-loss metallic state at elevated temperatures, experiencing a thin film phase transition and stabilization. During this transition, the VO_2_ crystal structure transforms from the monoclinic system to the diamond phase tetragonal system [36], as depicted in Figure 3.

In the simulation, the variable frequency complex permittivity of VO_2_ in the terahertz band can be described by the Drude model $\varepsilon_{VO_{2}}(\omega )=\varepsilon_{\infty }-\frac{\omega_{p}^{2}(\sigma )}{\omega^{2}+i\gamma_{VO_{2}}\omega }$ [37], where $\varepsilon_{\infty }=12$ is the permittivity of VO_2_ at the high frequency limit, $\sigma =3\times 10^{5}$ S/m is the conductivity when VO_2_ is converted to a stable metallic state, $\omega_{p}(\sigma )=1.4\times 10^{15}$ rad/m is the plasma frequency of VO_2_, and $\gamma_{VO_{2}}=5.75\times 10^{13}$ rad/m is the collision frequency [43,44]. When VO_2_ is in the insulating and metallic states, the conductivity is set to 30 S/m and 3 × 10^5^ S/m, respectively. Similarly, the metal gold of the metasurface can be described by the Drude model $\varepsilon_{Au}(\omega )=1-\frac{\omega_{p}^{2}}{\omega^{2}+i\gamma_{Au}\omega }$ [37], where $\omega_{p}=1.37\times 10^{16}$ Hz is the plasma frequency of Au, and the collision frequency is set as $\gamma_{Au}=4.08\times 10^{13}$ Hz.

In the THz band, the surface conductivity of graphene $\sigma_{\mathrm{gra}}$ can be described using the Drude model [45]:

(1) $$\sigma_{\mathrm{gra}}\approx \frac{e^{2}\mu_{c}}{\pi \hbar^{2}}\frac{j}{\omega +j/\pi }$$

(2) $$\mu_{c}\approx \hbar \upsilon_{f}\sqrt{\frac{\pi \varepsilon_{0}\varepsilon_{r}V_{g}}{et}}$$

where $\mu_{c}$, ℏ, and $V_{g}$ are the Fermi level, reduced Planck constant, and excitation voltage, respectively; $\varepsilon_{0}$ and $\varepsilon_{r}$ are the permittivity of the vacuum and substrate; and $\upsilon_{f}=1.1\times 10^{6}$ m/s is the Fermi velocity. Therefore, it can be inferred that by applying an excitation voltage, $\sigma_{\mathrm{gra}}$ will undergo modulation, thus tuning the performance of the designed metasurface.

In addition, the designed metasurface structure takes into account a predetermined manufacturing scheme. For the preparation of the copper substrate, the polyimide film is cleaned with acetone at room temperature, followed by ion cleaning and drying. The treated polyimide film then serves as a substrate, onto which a copper film with a thickness of 2 µm is deposited using magnetron sputtering [46]. Graphene is grown on Cu through chemical vapor deposition and transferred onto the sample using the poly methyl methacrylate transfer technique. A layer of poly methyl methacrylate is spin-coated onto the graphene on Cu, acting as a carrier for the graphene, and placed in an ammonium persulfate solution for 3 h to remove the copper foil. Then, the stacked poly methyl methacrylate/graphene is scooped from the water onto the desired substrate, followed by cleaning with iso-propanol to remove the poly methyl methacrylate [18]. Finally, the VO_2_ film can be deposited on the substrate through magnetron sputtering [30].

## 3. Results and Discussions

### 3.1. Multitasking Operating Modes

**Figure 4:**
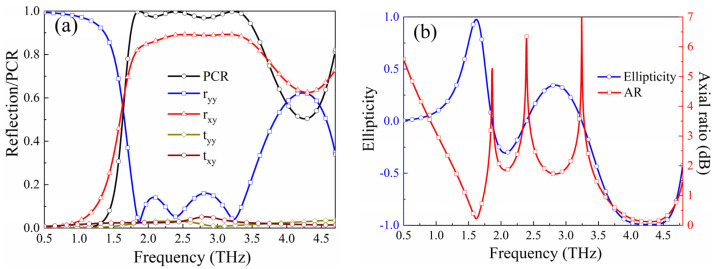
The simulated results of reflection polarization conversion mode: (**a**) PCR and S parameters; (**b**) ellipticity and axial ratio.

For the reflection-type polarization conversion mode, the performance is demonstrated in Figure 4. Figure 4a plots the S parameters and the Polarization Conversion Ratio (PCR); due to the extremely small transmission coefficients *t_yy_* and *t_xy_*, *PCR* can be defined accordingly: *PCR* = |*r_xy_*|^2^/(|*r_xy_*|^2^ + |*r_yy_*|^2^), where *r_xy_* and *r_yy_* are the cross-polarization reflection coefficient and the co-plane polarization reflection coefficient, respectively. As observed in Figure 4a, *r_xy_* approaches 0.9, and *r_yy_* closely tends to 0.1 in the range of 1.75–3.55 THz, resulting in the PCR exceeding 90% in this band. Additionally, *r_xy_* and *r_yy_* are approximately equal in the range of 3.60–4.69 THz, suggesting that the co-plane polarization EM waves are converted into circular polarization. Figure 4b illustrates the corresponding ellipticity and axial ratio (AR) based on the Stokes parameters [47]. It can be seen that within the circular polarization conversion operating band, the ellipticity is close to −1, and the AR is less than 1 dB, confirming that the co-polarized EM waves incident on the metasurface in the 3.60–4.69 THz are converted into RHCP.

**Figure 5:**
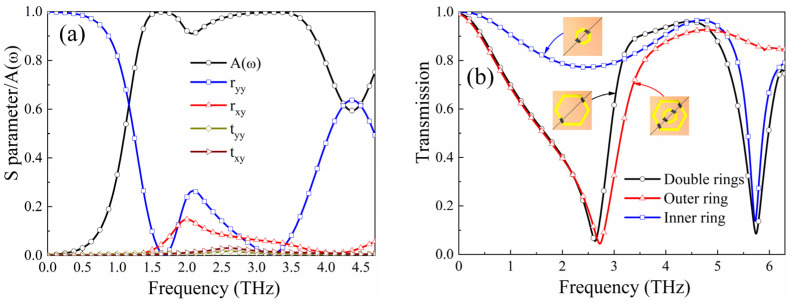
(**a**) Absorptivity and S parameters of the absorption mode; (**b**) transmission coefficients of the EIT-like mode.

Figure 5 presents the simulation results for both the absorption mode and EIT-like mode. The absorptivity utilized as a metric to evaluate EM shielding performance can be defined by *A*(ω) = 1 − *R*(ω) − *T*(ω), where *R*(ω) and *T*(ω) are the reflectivity and transmissivity, respectively. Given that the bottom layer of the designed metasurface is BPL, which exhibits a metallic phase at high temperatures and can block most of the EM waves, the formula can be modified to *A*(ω) = 1 − *R*(ω) = 1 − |*r_yy_*|^2^ − |*r_xy_*|^2^. As observed in Figure 5a, in the range of 1.34–3.82 THz, both *r_yy_* and *r_xy_* are less than 0.3, thereby achieving an absorptivity of over 90%. Figure 5b illustrates the transmission coefficients when the incentive conditions are switched to low temperature, and the metasurface operates in the transmission-type EIT-like mode. To elucidate the contribution of the nested hexagonal resonators in PML to the EIT-like performance, the transmission coefficients for each hexagonal resonator are plotted separately. It is noticeable that the outer ring exhibits a resonant frequency of 2.59 THz, whereas the inner ring’s resonant frequency occurs at 5.74 THz. Both hexagonal ring junctions can resonate with strong interaction with EM waves; thus, they can be considered as the resonance state of two bright modes. When both rings are combined, an EIT-like structure with a bright–bright mode is generated. From Figure 5b, a transparent window with a transmission coefficient greater than 0.9 can be observed in the range of 3.48–4.93 THz. Therefore, the two hexagonal structures, directly excited by the incident wave, produce strong destructive interference through near-field coupling and create a noticeable transmission window near a specific frequency, which is a typical EIT-like phenomenon.

### 3.2. Performance Regulation

**Figure 6:**
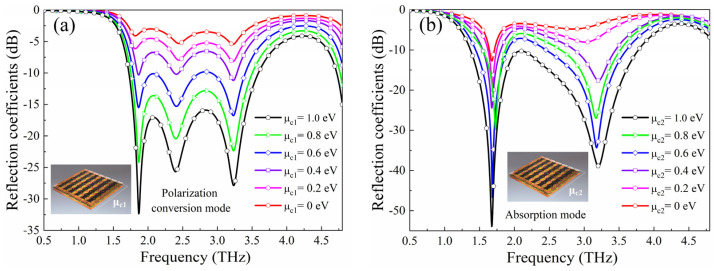
The electrical control for (**a**) reflection polarization conversion mode and (**b**) absorption.

The operation performance of the designed metasurface can be controlled by external excitation conditions, such as temperature, voltage, and polarization direction. Figure 6 illustrates the tuning capabilities of the applied bias voltage on the polarization conversion mode and the absorption mode. As observed in Figure 6a, when the bias voltage applied to the PML is gradually reduced from 1 eV to 0 eV, the co-plane polarization reflection coefficient *r_yy_* increases from below −15 dB to around −5 dB, indicating that with the reduction of the bias voltage, the co-plane polarization reflection coefficient is no longer converted but reflected in the original state. Similarly, as seen in Figure 6b, when the bias voltage applied to the ESL is gradually decreased, the reflection coefficient of the absorption mode also increases, resulting in less effective absorption of EM waves. Notably, the reflection coefficient remains minimal near the low frequency of 1.41 THz, which is attributed to the contribution of the PML for the absorption mode. This will be detailed in subsequent sections. Therefore, through the above analysis, it can be concluded that the operational performance of the proposed metasurface can be adjusted by the bias voltage, achieving tunable performance.

**Figure 7:**
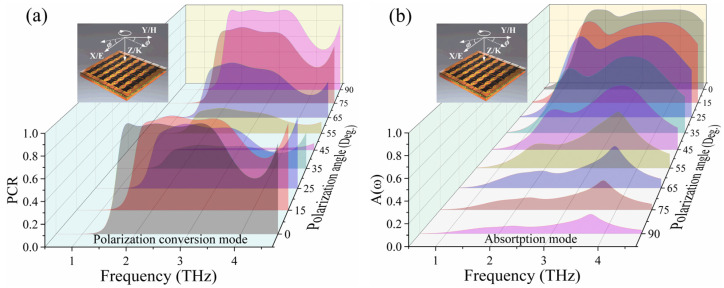
The polarization control for (**a**) reflection polarization conversion mode and (**b**) absorption.

Additionally, the anisotropic properties of both the ESL and PML in the design enable distinct responses to EM waves depending on their polarization direction. Therefore, the performance of the metasurface can be modulated through the polarization angle *ϕ*. Figure 7 illustrates the performance of the polarization conversion mode and the absorption mode at different polarization angles. As observed in Figure 7a, when the polarization angle increases from 0° to 45°, The polarization conversion performance gradually decreases. However, as the polarization angle gradually increases from 45° to 90°, the PCR returns to its original state. This is because the designed resonator in the PML is symmetrically oriented at 45° to the x-axis, resulting in the polarization conversion performance being turned off at a polarization angle of 45° while maintaining good performance at polarization angles of 0° and 90°. For the absorption mode, due to the hourglass-shaped resonators in the ESL being smaller in the middle and larger on the sides, at a polarization angle of 0°, EM waves can respond with the longer side *L*_2_, leading to EM wave energy dissipation within the metasurface, whereas the shorter side *L*_1_ of the resonator does not respond with the EM waves in this frequency range. When the polarization angle increases to 90°, the absorption performance almost disappears, as shown in Figure 7b. Thus, the designed structure can modulate its operation performance through either bias voltage or polarization direction.

### 3.3. Operating Mechanism

**Figure 8:**
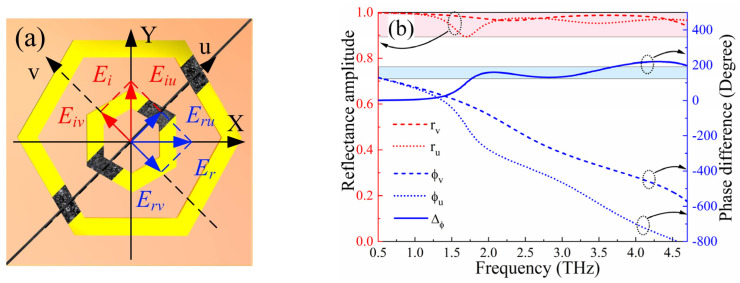
(**a**) Schematic diagram of the polarization conversion mechanism; (**b**) amplitudes, phases, and phase differences of the reflection coefficients.

The operating mechanism of the reflection-type polarization conversion mode can be elucidated using orthogonal eigenmodes, as depicted in Figure 8. In the simulations, EM waves are incident upon the metasurface with a polarization aligned along the Y-axis. Consequently, the incident and reflected electric fields can be decomposed into two components ${\vec{E}}_{i}=\vec{u}E_{iu}e^{j\phi }+\vec{v}E_{iv}e^{j\phi }$ and ${\vec{E}}_{r}=\vec{u}r_{u}E_{iu}e^{j(\phi +\phi_{u})}+\vec{v}r_{v}E_{iv}e^{j(\phi +\phi_{v})}$, oriented along the u-axis and v-axis, respectively. Here, $r_{u}$ and $r_{v}$ are the reflection amplitudes along these axes, while $\phi_{u}$ and $\phi_{v}$ denote their associated phases. Owing to the anisotropic characteristics of the nested hexagonal resonators in the design, a phase difference $\Delta \phi_{vu}$ is introduced between the reflected and incident wave phases. For the LTL mode, should conditions $r_{u}\approx r_{v}$ and $\Delta \phi_{vu}\approx \pm \pi$ be satisfied concurrently, the electric field vector of the incident EM waves will experience a 180° rotation upon interaction with the metasurface, thereby facilitating efficient LTL performance. Figure 8 presents the simulation outcomes for incident EM waves polarized along the u-axis and v-axis. As shown in Figure 8b, within the LTL operation frequency band of 1.75–3.55 THz, parameters $r_{u}$ and $r_{v}$ remain largely equivalent, and the phase disparity approximates 180°. This finding underscores that the devised structure exhibits commendable polarization conversion efficiency.

In considering the absorption mode, one can analyze the absorption mechanism by determining the equivalent impedance, permittivity, and permeability of the metasurface using the S-parameter retrieval method [48]:

(3) $$n(\omega )=\frac{1}{kd}\cos^{-1}\left[\frac{1}{2S_{21}}(1-S_{11}^{2}+S_{21}^{2})\right]$$

(4) $$Z(\omega )=\pm \sqrt{\frac{{\left(1+S_{11}\right)}^{2}-S_{21}^{2}}{{\left(1-S_{11}\right)}^{2}-S_{21}^{2}}}$$

**Figure 9:**
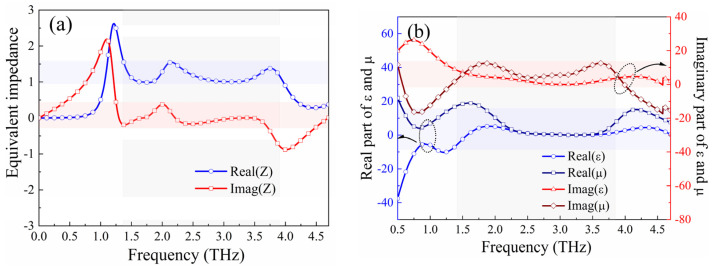
(**a**) Normalized equivalent impedance and (**b**) equivalent permittivity and permeability.

(5) ε(ω)=n(ω)/Z(ω)

(6) μ(ω)=n(ω)Z(ω)

where k is the wave number, and d corresponds to the thickness of the medium layer involved in the absorption mode. When EM waves respond with the metasurface and the normalized equivalent impedance matches the impedance $Z_{0}$ in free space, $Z(\omega )=\sqrt{\mu (\omega )/\varepsilon (\omega )}\approx Z_{0}=1$, the reflection of EM waves by the metasurface is minimized, thereby maximizing the absorptivity. Figure 9 illustrates the normalized equivalent impedance, permittivity, and permeability of the metasurface derived through the S-parameter retrieval method. As observed in Figure 9a, within the operation frequency band of the absorption mode, 1.34–3.82 THz, the real part of the impedance approaches 1, and the imaginary part is close to 0. Figure 9b shows that, in this frequency range, the imaginary parts of the permittivity and permeability are near 0, and their real parts are approximately equal, achieving favorable impedance matching conditions. This demonstrates that the proposed metasurface can absorb EM waves into the material, where they are then dissipated by the material’s losses, thus achieving the desired absorptive performance.

To demonstrate the effectiveness of EIT-like phenomena in the designed metasurface, a coupled oscillator model is employed for analysis. The resonance within the nested hexagonal rings and the BPL layer in the metasurface is excited by $E(t)=E_{0}e^{-i\omega t}$. The coupling equations representing their interactions are expressed as follows [49]:

(7) $$\left\{\begin{array}{l} {\ddot{x}}_{1}(t)+\gamma_{1}{\dot{x}}_{1}(t)+\omega_{1}^{2}x_{1}(t)+\kappa^{2}x_{2}(t)=\frac{g_{1}E}{m_{1}}\\ {\ddot{x}}_{2}(t)+\gamma_{2}{\dot{x}}_{2}(t)+\omega_{2}^{2}x_{2}(t)+\kappa^{2}x_{1}(t)=\frac{g_{2}E}{m_{2}} \end{array}\right.$$

where $x_{1}$ and $x_{2}$ are the complex amplitudes of the two bright modes; ($g_{1}$,$g_{2}$), ($m_{1}$,$m_{2}$), ($\omega_{1}$,$\omega_{2}$), and ($\gamma_{1}$,$\gamma_{2}$) are the effective charges, effective masses, resonant angular frequencies, and modal loss factors, respectively. κ is the coupling strength between the two bright modes. *A* and *B* are dimensionless constants used to describe the relative coupling of the incident wave, defined as $A=g_{1}/g_{2}$ and $B=m_{1}/m_{2}$. $\chi =P/\varepsilon_{0}E$ is an expression of susceptibility, where $P=g_{1}x_{1}+g_{2}x_{2}$ is the polarization of the particle. Substituting $x_{1}$ and $x_{2}$ into the expression for χ yields the following:

(8) $$\chi =\frac{K}{A^{2}B}\cdot \left(\frac{A(B+1)\kappa^{2}+A^{2}((\omega^{2}-\omega_{2}^{2})+B(\omega^{2}-\omega_{1}^{2}))}{\kappa^{4}-(\omega^{2}-\omega_{1}^{2}+i\omega \gamma_{1})(\omega_{2}-\omega_{2}^{2}+i\omega \gamma_{2})}+i\omega \frac{A^{2}\gamma_{1}+B\gamma_{2}}{\kappa^{4}-(\omega^{2}-\omega_{1}^{2}+i\omega \gamma_{1})(\omega^{2}-\omega_{2}^{2}+i\omega \gamma_{2})}\right)$$

**Figure 10:**
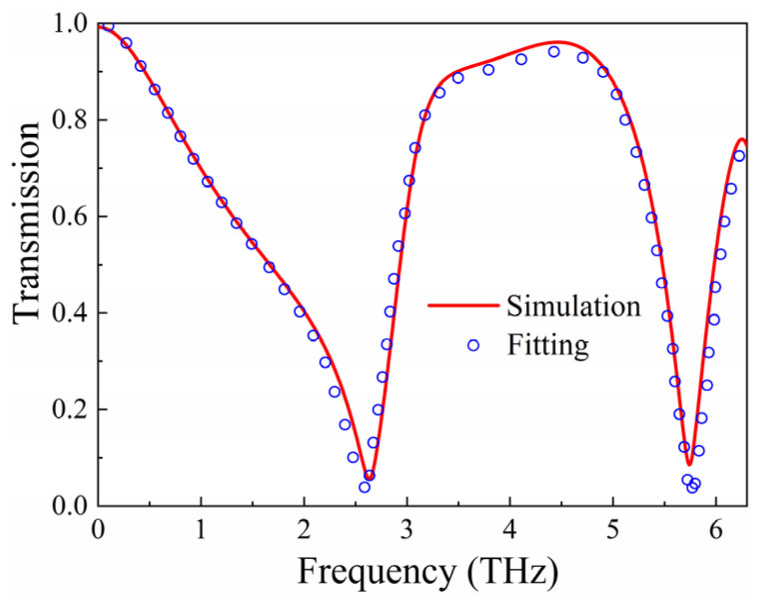
Transmission results for EM simulated and theoretical fitting.

In the process of numerical fitting, the selected parameters are $\omega_{1}$ = 2.59 THz, $\omega_{2}$ = 5.72 THz, *A* = 3.91, *B* = 2.26, κ = 0.25, $\gamma_{1}$ = 2.5 × 10^11^ rad/s, and $\gamma_{2}$ = 2.6 × 10^11^ rad/s. Figure 10 illustrates the transmission results from both EM simulations and theoretical fitting. It can be observed from the figure that there is a good agreement between them.

**Figure 11:**
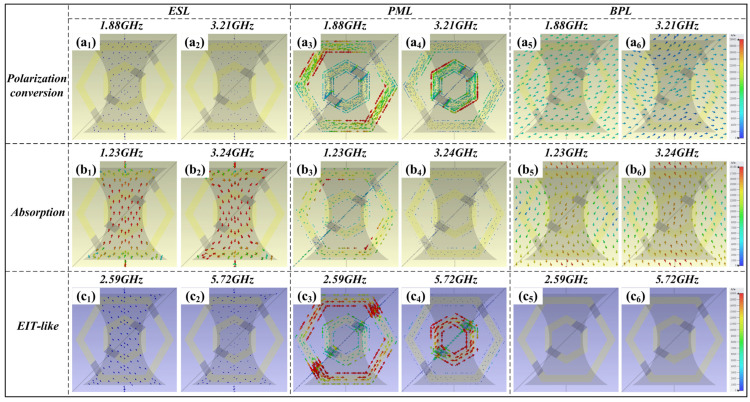
Surface current distribution for (**a_1_**–**a_6_**) polarization conversion mode; (**b_1_**–**b_6_**) absorption mode and (**c_1_**–**c_6_**) EIT-like mode.

The surface current distribution of the metasurface under design sheds light on its underlying operational mechanisms for various functional tasks. Figure 11 presents the surface current distribution across each layer of the metasurface at the corresponding resonant frequency. In the reflection-type polarization conversion mode, the surface current is notably weak in the ESL but intensifies within the nested hexagonal resonators of the PML and the BPL, as observed in Figure 11(a_1_–a_6_). This suggests that the polarization conversion mechanism is predominantly driven by the EM response between the PML and the BPL, wherein the current directions on the hexagonal rings are approximately opposite to those on the BPL, implying magnetic resonance phenomena. Furthermore, it is discernible that the lower frequency resonance at 1.88 THz primarily arises from the outer ring, while the higher frequency resonance at 3.21 THz is largely attributable to the inner ring. During the absorption mode, the surface current concentrates mainly on the hourglass-shaped graphene resonators in the ESL and the BPL, as shown in Figure 11(b_1_–b_6_). This concentration indicates that the graphene resonators play a crucial role in the absorptive mode’s resonant behavior. Moreover, a significant current distribution is evident on the hexagonal outer ring resonators within the PML, as depicted in Figure 11b_3_, pointing to a hybrid resonance at the lower frequency of 1.23 GHz, induced by both the graphene resonators in the ESL and the hexagonal outer ring resonators in the PML. This observation clarifies why the metasurface maintains some level of absorption even when the graphene resonators’ feeding voltage *μ*_c2_ is set to zero, as illustrated in Figure 6, given that the hexagonal outer ring resonators persistently contribute to the absorption performance. For the EIT-like mode, as shown in Figure 11c, the current density is largely confined to the hexagonal resonators of the PML, with the current at the lower frequency of 2.59 GHz being mostly associated with the hexagonal outer ring resonators, and the current at the higher frequency of 5.72 GHz primarily linked to the hexagonal inner ring resonators-aligning with the findings discussed in Figure 5b.

Finally, we compared the proposed metasurface results with those recently reported studies in terms of functionality, operational mode, and excitation materials, as shown in Table 3. It can be seen that most works focus on polarization conversion and absorption functions in reflection mode, while metasurfaces with transmission capabilities often lack tunable performance for EM waves. In contrast, the proposed metasurface not only operates in reflection, transmission, and absorption modes but also fully adjusts the polarization characteristics of EM waves. Additionally, its performance can be regulated by graphene and VO_2_. Therefore, the metasurface proposed in this paper holds advantages in terms of multi-mode and multifunctional.

## 4. Conclusions

This study presents a metasurface that boasts reflection, transmission, and absorption functionalities. Its design is rooted in a dual-layered architecture that integrates VO_2_ and graphene as adjustable elements. By exposing the metasurface to diverse excitation conditions, it can be switched between various operation modes tailored to specific tasks. For the reflection-type polarization conversion task, the metasurface converts co-planar polarization EM waves to cross-polarization counterparts in the range of 1.75–3.55 THz, while linearly polarized waves are converted to circular polarization waves in the range of 3.60–4.69 THz. When in absorption mode, EM waves are entirely absorbed in the 1.34–3.82 THz, achieving perfect shielding. In the EIT-like mode, a distinctive transmission window materializes within the 3.48–4.93 THz. Moreover, this study discusses modulating the metasurface’s performance by altering the bias excitation voltage and polarization direction. A comprehensive analysis of the intrinsic working principles underlying this metasurface is provided, drawing upon eigenmodes, S-parameter extraction methods, oscillator coupling model, and current distribution patterns. Such a meticulously engineered metasurface paves the way for the creation of multifaceted devices that seamlessly amalgamate reflection, transmission, and absorption.

## Figures and Tables

**Table 1:** Multitasking operating modes of the proposed metasurface under different excited conditions.

Operating Mode	Excited Conditions	Frequency (in THz)
Polarization conversion	High temperature *μ*_c1_ = 1 eV, *μ*_c2_ = 1 eV	Co-planar to cross-polarization (LTL): 1.75–3.55 THz
		Co-planar to circular polarization (RHCP): 3.60–4.69 THz
Absorption	High temperature *μ*_c1_ = 0 eV, *μ*_c2_ = 0 eV	1.34–3.82 THz
EIT-like	Low temperature *μ*_c1_ = 1 eV, *μ*_c2_ = 0 eV	3.48–4.93 THz

**Table 2:** Optimized structure parameters of the proposed metasurface (in μm).

*H*_1_	*H*_2_	*P*	*L*_1_	*L*_2_	*R*_1_	*R*_2_	*R*_3_	*W*_1_	*W*_2_
9.88	6.03	26.60	20.34	22.0	13.11	12.02	6.02	1.71	3.12

**Table 3:** Comparisons of the main results with previous studies.

Ref.	Functionality	Operation Mode	Excitation Material
[25]	LTL polarization conversion; Absorption	Reflection	Photoconductance Si
[34]	Single, dual, broadband, and ultra-broadband absorption	Reflection	VO_2_; Graphene
[35]	LTL polarization conversion; Absorption	Reflection	VO_2_; Graphene
[37]	LTL and LTC polarization conversion	Reflection	VO_2_; Graphene
[42]	Broadband absorption; Transmission	Absorption; Transmission	VO_2_
This work	LTL and LTC polarization conversion; Absorption; EIT-like	Reflection; Absorption; Transmission	VO_2_; Graphene

## Data Availability

The original contributions presented in the study are included in the article, further inquiries can be directed to the corresponding author.
